# Substituting prolonged sedentary time and cardiovascular risk in children and youth: a meta-analysis within the International Children’s Accelerometry database (ICAD)

**DOI:** 10.1186/s12966-019-0858-6

**Published:** 2019-10-31

**Authors:** Katrien Wijndaele, Thomas White, Lars Bo Andersen, Anna Bugge, Elin Kolle, Kate Northstone, Niels Wedderkopp, Mathias Ried-Larsen, Susi Kriemler, Angie S. Page, Jardena J. Puder, John J. Reilly, Luis B. Sardinha, Esther M. F. van Sluijs, Stephen J. Sharp, Søren Brage, Ulf Ekelund, On behalf of the International Children’s Accelerometry Database (ICAD) Collaborators, L. B. Andersen, A. J. Atkin, G. Cardon, R. Davey, U. Ekelund, D. W. Esliger, P. Hallal, B. H. Hansen, K. F. Janz, S. Kriemler, N. Møller, K. Northstone, A. Page, R. Pate, J. J. Puder, J. J. Reilly, J. Salmon, L. B. Sardinha, L. B. Sherar, A. Timperio, E. M. F. van Sluijs

**Affiliations:** 1 MRC Epidemiology Unit, Institute of Metabolic Science, University of Cambridge, Box 285, Addenbrooke’s Hospital Hills Road Cambridge, Cambridge, CB2 0QQ UK; 2 Faculty of Teacher Education and Sport, Campus Sogndal, Western Norway University of Applied Sciences, Bergen, Norway; 3 Department of Sport Medicine, Norwegian School of Sport Sciences, Oslo, Norway; 4 Centre for Research in Childhood Health, Exercise Epidemiology Unit, Department of Sport Sciences and Clinical Biomechanics, University of Southern Denmark, Odense, Denmark; 5 Physiotherapy, University College Copenhagen, Copenhagen, Denmark; 6 Bristol Medical School, University of Bristol, Bristol, UK; 7 Institute of Social and Preventive Medicine, University of Zurich, Zürich, Switzerland; 8 Centre for Exercise, Nutrition and health Sciences, School for Policy Studies, University of Bristol, Bristol, UK; 9 Lausanne University Hospital, Lausanne, Switzerland; 10 School of Psychological Sciences and Health, University of Strathclyde, Glasgow, Scotland; 11 Exercise and Health Laboratory, CIPER, Faculdade de Motricidade Humana, Universidade de Lisboa, Lisbon, Portugal; 12 UKCRC Centre for Diet and Activity Research (CEDAR), University of Cambridge, Cambridge, UK

**Keywords:** ICAD, ALSPAC, Prolonged sitting, Cardio-metabolic, Iso-temporal, Physical activity

## Abstract

**Background:**

Evidence on the association between sitting for extended periods (i.e. prolonged sedentary time (PST)) and cardio-metabolic health is inconsistent in children. We aimed to estimate the differences in cardio-metabolic health associated with substituting PST with non-prolonged sedentary time (non-PST), light (LIPA) or moderate-to-vigorous physical activity (MVPA) in children.

**Methods:**

Cross-sectional data from 14 studies (7 countries) in the International Children’s Accelerometry Database (ICAD, 1998–2009) was included. Accelerometry in 19,502 participants aged 3–18 years, together with covariate and outcome data, was pooled and harmonized. Iso-temporal substitution in linear regression models provided beta coefficients (95%CI) for substitution of 1 h/day PST (sedentary time accumulated in bouts > 15 min) with non-PST, LIPA or MVPA, for each study, which were meta-analysed.

**Results:**

Modelling substitution of 1 h/day of PST with non-PST suggested reductions in standardized BMI, but estimates were > 7-fold greater for substitution with MVPA (− 0.44 (− 0.62; − 0.26) SD units). Only reallocation by MVPA was beneficial for waist circumference (− 3.07 (− 4.47; − 1.68) cm), systolic blood pressure (− 1.53 (− 2.42; − 0.65) mmHg) and clustered cardio-metabolic risk (− 0.18 (− 0.3; − 0.1) SD units). For HDL-cholesterol and diastolic blood pressure, substitution with LIPA was beneficial; however, substitution with MVPA showed 5-fold stronger effect estimates (HDL-cholesterol: 0.05 (0.01; 0.10) mmol/l); diastolic blood pressure: − 0.81 (− 1.38; − 0.24) mmHg).

**Conclusions:**

Replacement of PST with MVPA may be the preferred scenario for behaviour change, given beneficial associations with a wide range of cardio-metabolic risk factors (including adiposity, HDL-cholesterol, blood pressure and clustered cardio-metabolic risk). Effect estimates are clinically relevant (e.g. an estimated reduction in waist circumference of ≈1.5 cm for 30 min/day replacement). Replacement with LIPA could be beneficial for some of these risk factors, however with substantially lower effect estimates.

## Background

Maintenance of sufficient levels of moderate-to-vigorous physical activity (MVPA) is a well-accepted lifestyle target for optimal health and development in young people [1]. Sedentary time, i.e. any waking behaviour with low energy expenditure, while in sitting/reclining/lying posture [2], has recently gained attention as a potential additional health risk factor, and is highly prevalent, even in children [3, 4]. Lower fitness levels and unfavourable cardiovascular risk profiles have been associated with excessive self-reported TV viewing, independent of MVPA [5]. For accelerometry-assessed total sedentary time, such associations are less consistent in children and adolescents [1, 3–5].

Total sedentary time is, however, accumulated in varying patterns. Some tend to accumulate predominantly long sedentary bouts whereas others display more regular interruptions, while still accruing similar volumes of total sedentary time [6]. These diverse accumulation patterns may have different associations with health indicators, which cannot be detected by only examining total volume of sedentary time. In adults, evidence from experimental and observational research indicates a protective effect of regular interruptions in sitting time on specific cardio-metabolic biomarkers, including adiposity, glucose metabolism and inflammation, even when these interruptions are only of light intensity [6]. In paediatric populations, far fewer studies have examined this. Recent reviews in this age group concluded that the current evidence on associations between objectively measured accumulation patterns of sedentary time and cardio-metabolic health is inconsistent [3–5, 7]. These reviews could not include meta-analyses due to methodological differences among contributing studies [3–5, 7]. Despite this low level of evidence, some national public health authorities have implemented recommendations for paediatric populations to limit sitting time for “extended” periods (i.e. prolonged sedentary time (PST)) [8, 9].

If PST were cardio-metabolically harmful, guidelines designed to minimise PST should be fully informed by the relative merits of replacing it with other activity behaviours. Total awake time in the day is finite and the impact of reductions in PST on health depends on what behaviour it is displaced with, whether this is non-prolonged sedentary time (non-PST), light-intensity physical activity (LIPA) or MVPA [6]. Some of these activity subcomponents may be more amenable to behaviour change than others [10], further reinforcing the need to examine the potential impact of these different substitution scenarios. This is however largely unexplored in children and adolescents using device-based measures [5], certainly on a sufficiently large scale to allow for full examination of differences in such substitution effects by sex and age.

Using the International Children’s Accelerometry Database (ICAD), containing pooled accelerometry and cardio-metabolic health data from an extensive number of studies in children and adolescents worldwide, we aimed to examine 1) the independent associations between PST and cardio-metabolic health, as well as 2) the associations following iso-temporal substitution modelling of replacing PST with other activity, using a meta-analytical approach on harmonized data [11]. Such insights are important to guide intervention design and strengthen the evidence-base underpinning public health guidelines in this age group.

## Methods

### Participants

Data from 21 studies in children aged 3–18, with, as a minimum, objectively measured activity and sedentary time by a waist-worn accelerometer (Actigraph Corp., Pensacola, FL, USA), as well as sex, age and measured height and weight, were pooled in the ICAD. A detailed description of the ICAD aims, design, study characteristics and covariate measurement has previously been published [11]. Participants of 14 of the 21 studies which provided information on at least one cardio-metabolic variable, more specifically waist circumference (the most commonly measured), constituted the sampling frame for the current cross-sectional analysis (*n* = 20,296). After excluding those individuals with insufficient valid accelerometry data (*n* = 788) and invalid data for some of the outcome measures (*n* = 6), a total sample of 19,502 children were included in analyses. The study complies to the Declaration of Helsinki, all study protocols were approved by local ethics committees and informed consent was obtained from all subjects or their legal authorized representative.

### Anthropometric and cardio-metabolic risk variables

Height, weight and waist circumference were measured in all 14 included studies. Height and weight were measured objectively using standardized methods, and used to derive body mass index (BMI; weight(kg)/(height(m)^2^)). Age- and sex-specific BMI standard deviation scores (BMI z-scores) were calculated based on the LMS method [12]. Waist circumference was measured by metal anthropometric tape just above the iliac crest in one study [13], and halfway between the lower rib margin and the iliac crest in the other studies, following gentle expiration [14–22] (on the skin in all studies, except two that allowed light clothing [17, 20]). Due to the difference in measurement method between studies and the lack of reference data covering the entire age range of the ICAD sample, waist circumference was not standardized, similar to previous ICAD analyses [1].

Ten studies provided measurements of systolic and diastolic blood pressure, as described in detail elsewhere [1, 15, 18, 19, 22–25]. Fasting triglycerides and HDL-cholesterol levels were assessed in 8 studies [15, 16, 22–24, 26], and fasting glucose and insulin in 7 studies [15, 16, 22–24] using standardized clinical procedures [1]. Six participants with unrealistic values for fasting glucose and insulin were excluded from analyses.

A clustered cardio-metabolic risk score (CCMR) was calculated based on indicators of central adiposity (waist circumference), lipid (triglycerides and HDL-cholesterol) and glucose (fasting glucose and insulin) metabolism and blood pressure (systolic and diastolic blood pressure) [27]. Sex-specific standardization of each individual variable was based on the mean and SD of all boys and girls without missing data for the respective variable. Triglycerides and insulin values were first normalized (log 10), due to their skewed distribution. After averaging systolic and diastolic blood pressure and inverting HDL-cholesterol z-scores, CCMR was calculated by summing individual z-scores and dividing by 6 (i.e. the number of contributing components). The same score was also calculated without the central adiposity component (CCMR_no adip_) for models with additional adjustment for waist circumference [27]. These risk scores were calculated for participants with data on all contributing cardio-metabolic variables.

### Objectively measured sedentary time and physical activity

All accelerometry data were processed centrally using open-source software [28]. Higher resolution files (epoch length < 60 s) were reintegrated to 60 s epoch resolution and all time between midnight and 7 am was discarded. All epochs with an intensity > 30,000 counts per minute (cpm) were classified as non-valid. Non-wear time was defined as bouts lasting ≥60 min of consecutive zeros. Children with ≥1 valid day, each containing ≥500 min of monitor wear time (from 7 am to midnight), passed the inclusion criteria (*n* = 19,695). Out of these, all files previously deemed invalid (*n* = 201) [11], as well a small number of additional files generated by mechanically faulty monitors (*n* = 14) were excluded. The latter 14 cases were verified as corrupt data via visual screening of files, identified due to implausible plateaus at low or high acceleration levels.

All valid wear time registering acceleration < 100 cpm was defined as total sedentary time. Cut-offs were ≥ 100 cpm and < 3000 cpm for LIPA, and ≥ 3000 cpm for time spent in MVPA [1]. PST was characterized as the duration of sedentary time accumulated in bouts > 15 min (PST > 15 min) and > 30 min (PST > 30 min) [29]. PST defined by minimal bout durations > 60 min showed minimal prevalence in this age group (< 1% of total sedentary time) and was therefore not considered. Non-PST of ≤15 min and ≤ 30 min was calculated as the difference between total sedentary and the respective PST volumes. All variables were expressed in h/day.

### Statistical analysis

Descriptive characteristics were calculated for the total sample and separately by sex (mean (standard deviation (SD)) for normally distributed, medians (interquartile range (IQR)) for non-normally distributed and n (%) for categorical variables). Spearman correlations were calculated between sedentary and activity time components.

Linear regression models were fit within each study to estimate associations between PST and the cardio-metabolic risk factors. For each of the exposure-outcome combinations, multi-collinearity (variance inflation factor), homoscedasticity (plot of residuals versus fitted values) and normality of residuals (histogram) were checked in the largest study with greatest variability in age, ethnicity, and cardio-metabolic variables measured [23]. Models were initially adjusted for sex, age (years) and monitor wear time (hours/day; Model A). Subsequently, we added MVPA time (Model B) and waist circumference (Model C) to examine independence of these covariates. Random effects meta-analysis was used to derive a pooled regression coefficient (95% CI) across studies. Sex interactions were examined within each study in Model A and meta-analysed. Age (< 12 versus ≥12) interactions were examined, within each study with an age range incorporating 12 years (≥5 studies, depending on outcome), and meta-analysed. Age interactions were examined in the total group when no sex interaction was found and separately within boys and girls when a significant sex interaction was found.

We then examined iso-temporal substitution [30] of PST with non-PST, LIPA and MVPA for those outcome variables which showed a significant association with PST in Model A described above, providing results for the total group where no sex interaction was found and additionally for both sexes separately where a sex interaction was found. The iso-temporal substitution models within each study included sex (where applicable), age, wear time, non-PST, LIPA and MVPA. Regression coefficients (95% CI) of the included activity components in these models provide an estimate of the change in the outcome variable when increasing that type of activity by 1 h/day while decreasing PST by the same duration and holding other activity components constant [30], and were also meta-analysed.

A first set of sensitivity analyses was conducted in a smaller set of children who provided ≥3 valid days of accelerometry data, including ≥1 weekend day. A second set of sensitivity analyses was conducted, adjusting for a more comprehensive set of confounding variables, more specifically ethnicity (white/non-white), parental socio-economic status (SES; household income (categorical)), birth weight (kg) and sexual maturity (breast development in girls and pubic hair in boys, Tanner), where available. Due to missing data for these covariates, these analyses were performed in smaller samples. As this was an exploratory analysis of observational data and not a confirmatory analysis of a clinical trial, we did not correct for multiple testing. All analyses were conducted using Stata version 14 (*Stata Statistical Software.* College Station, TX: StataCorp LP. 2015) and statistical significance was set at *p* < 0.05.

## Results

### Descriptive characteristics

Characteristics of all included participants are provided in Table 1. The mean (SD) contribution to total sedentary time was 30.8% (12.9%) for PST > 15 min (boys: 30.3% (13.1%); girls: 31.3% (12.6%))) and 14.1% (10.7%) for PST > 30 min (boys: 13.9% (11.0%); girls: 14.2% (10.4%)). PST > 15 min and PST > 30 min were highly correlated (Table 2). Additional file 1: Table S1 provides individual cohort characteristics as well as descriptive statistics for the outcome variables within each cohort.

**Table 1:** Baseline descriptive characteristics of all participants and stratified by sex, *ICAD*

Characteristic^*^	Total *N* = 19,502	Boys *n* = 9440	Girls *n* = 10,062
Age, years	11.2 (2.7)	11.2 (2.7)	11.1 (2.7)
Weight, kg	42.5 (16.3)	42.7 (17.3)	42.3 (15.3)
Height, cm	146.2 (15.9)	146.9 (17.0)	145.5 (14.7)
Standardized body mass index	0.4 (1.2)	0.5 (1.2)	0.4 (1.2)
Waist circumference, cm	67.0 (12.1)	67.3 (12.3)	66.7 (11.9)
Triglycerides^†^, mmol/l	0.7 (0.5–1.0)	0.7 (0.5–0.9)	0.7 (0.5–1.0)
HDL-cholesterol, mmol/ l	1.5 (0.3)	1.5 (0.3)	1.5 (0.3)
Systolic blood pressure, mmHg	104.8 (10.6)	105.4 (10.9)	104.2 (10.3)
Diastolic blood pressure, mmHg	58.9 (8.5)	58.4 (8.7)	59.4 (8.3)
Fasting plasma glucose, mmol/ l	5.0 (0.5)	5.1 (0.5)	5.0 (0.5)
Fasting serum insulin^†^, pmol/ l	42.8 (27.0–65.3)	39.4 (25.0–59.0)	46.6 (29.9–71.1)
CCMR	−0.04 (0.61)	− 0.04 (0.63)	− 0.05 (0.59)
CCMR_*no adip*_	− 0.02 (0.61)	− 0.02 (0.62)	− 0.02 (0.60)
PST, > 15 min bouts^†^, hours/day	1.8 (1.1–2.6)	1.7 (1.1–2.5)	1.8 (1.2–2.7)
PST, > 30 min bouts^†^, hours/day	0.7 (0.4–1.2)	0.7 (0.3–1.2)	0.7 (0.4–1.3)
Non-PST, ≤15 min bouts, hours/day	4.1 (0.9)	4.1 (0.9)	4.2 (0.8)
Non-PST, ≤30 min bouts^†^, hours/day	5.3 (4.5–6.1)	5.2 (4.3–7.0)	5.4 (4.6–6.1)
Total sedentary time, hours/day	6.2 (1.6)	6.0 (1.6)	6.3 (1.6)
LIPA, hours/day	6.4 (1.4)	6.4 (1.4)	6.3 (1.4)
MVPA, hours/day	0.5 (0.4)	0.6 (0.4)	0.4 (0.3)
Monitor wear time, hours/day	13.0 (1.4)	13.1 (1.4)	13.0 (1.3)

Data are means (SD)

^*^ Collected in all participants, except for: standardized body mass index (boys: *n* = 9430, girls: n = 10,047), triglycerides (boys: *n* = 2625, girls: *n* = 2705), HDL-cholesterol (boys: *n* = 3818, girls: *n* = 4031), systolic (boys: *n* = 7177, girls: *n* = 7549) and diastolic blood pressure (boys: *n* = 7155, girls: *n* = 7532), fasting plasma glucose (boys: *n* = 2451, girls: *n* = 2501), serum insulin (boys: *n* = 2431, girls: *n* = 2481), CCMR and CCMR_*no adip*_(boys: *n* = 2276, girls: *n* = 2319)

^†^ Data are medians (IQR) due to skewed distribution

Abbreviations: *CCMR* clustered cardio-metabolic risk score including waist circumference, *CCMR*_*no adip*_ clustered cardio-metabolic risk score excluding waist circumference, *PST* prolonged sedentary time; LIPA: light-intensity physical activity, *MVPA* moderate-to-vigorous physical activity

**Table 2:** Spearman correlations between sedentary and activity time covariates

	PST, > 30 min bouts	Non-PST, ≤15 min bouts	Non-PST, ≤30 min bouts	Total sedentary time	LIPA	MVPA
PST, > 15 min bouts	0.87	0.20	0.54	0.84	− 0.67	− 0.32
PST, > 30 min bouts		− 0.01	0.23	0.64	− 0.52	− 0.26
Non-PST, ≤15 min bouts			0.87	0.64	−0.14	− 0.19
Non-PST, ≤30 min bouts				0.85	−0.41	−0.28
Total sedentary time					−0.59	−0.36
LIPA						0.33

Abbreviations: *PST* prolonged sedentary time, *Non-PST* non-prolonged sedentary time, *LIPA* light-intensity physical activity, *MVPA* moderate-to-vigorous physical activity

### Prolonged sedentary time and cardio-metabolic risk

More PST > 15 min was independently associated with higher standardized BMI, waist circumference, systolic and diastolic blood pressure and CCMR and lower HDL-cholesterol after adjustment for confounders in Model A (Table 3). Significant sex interactions were found for most outcomes, except standardized BMI. The associations of PST > 15 min with waist circumference, HDL-cholesterol, CCMR and CCMR_no adip_ were stronger in boys; there was a stronger positive association with diastolic blood pressure in girls. After adjustment for MVPA, most associations were attenuated and no longer significant, except for the inverse association with HDL-cholesterol in boys and the positive association with diastolic blood pressure in girls (Model B). The latter two associations were non-significant following further adjustment for waist circumference (Model C). There was no evidence that any of the PST > 15 min/outcome associations differed between age groups. Results for PST > 30 min were similar compared to those for PST > 15 min (Additional file 1: Table S2), which is commensurate with the fairly high correlation between both exposure variables (Table 2). Therefore only PST > 15 min was taken forward for further analyses. Sensitivity analyses in the subsample with ≥3 valid wear days including ≥1 valid weekend day showed comparable patterns of findings (Additional file 1: Table S3). Sensitivity analysis, allowing for more comprehensive confounding adjustment where available, showed somewhat smaller effect estimates compared to those in the main analyses (Additional file 1: Table S4).

**Table 3:** Cross-sectional associations between prolonged sedentary time and cardio-metabolic risk, ICAD

Outcome/N included	Model	PST, > 15 min bouts (h/day)			P sex interaction
		All	Boys	Girls	
Standardized BMI 19,477	A	**0.029 (0.001; 0.058)**	**–**	**–**	0.117
	B	0.00 (−0.02; 0.03)	–	–	
Waist circumference (cm) 19,502 (boys: 9440; girls: 10,062)	A	**0.30 (0.04; 0.56)**	**0.50 (0.16; 0.84)**	0.12 (−0.14; 0.38)	0.001
	B	0.10 (−0.11; 0.31)	0.22 (−0.05; 0.48)	− 0.01 (− 0.25; 0.24)	
Triacylglycerol (mmol/l) 5330	A	0.002 (− 0.000; 0.004)	**–**	**–**	0.233
	B	0.000 (−0.002; 0.002)	–	–	
	C	0.000 (−0.001; 0.002)	–	–	
HDL-cholesterol (mmol/l) 7849 (boys: 3918; girls: 4031)	A	**− 0.006 (− 0.011; − 0.000)**	**−0.014 (− 0.022; − 0.007)**	0.001 (− 0.007; 0.008)	0.001
	B	0.00 (− 0.01; 0.01)	**−0.008 (− 0.016; − 0.001)**	0.005 (− 0.002; 0.013)	
	C	− 0.00 (− 0.01; 0.00)	− 0.007 (− 0.015; 0.001)	0.003 (− 0.004; 0.011)	
Systolic blood pressure (mmHg) 14,726 (boys: 7177; girls: 7549)	A	**0.17 (0.02; 0.33)**	0.14 (− 0.04; 0.32)	0.20 (− 0.12; 0.52)	0.045
	B	0.05 (− 0.09; 0.18)	0.01 (− 0.18; 0.21)	0.07 (− 0.23; 0.37)	
	C	− 0.01 (− 0.13; 0.12)	− 0.06 (− 0.24; 0.12)	0.07 (− 0.14; 0.29)	
Diastolic blood pressure (mmHg) 14,687 (boys: 7155; girls: 7532)	A	**0.19 (0.09; 0.29)**	0.07 (− 0.07; 0.21)	**0.30 (0.17; 0.44)**	0.009
	B	0.09 (− 0.01; 0.20)	− 0.02 (− 0.17; 0.14)	**0.17 (0.03; 0.32)**	
	C	0.05 (−0.05; 0.15)	−0.07 (− 0.22; 0.08)	0.14 (− 0.01; 0.28)	
Fasting plasma glucose (mmol/l) 4952	A	0.005 (− 0.004; 0.014)	**–**	**–**	0.624
	B	−0.00 (− 0.01; 0.01)	–	–	
	C	−0.00 (− 0.01; 0.01)	–	–	
Fasting serum insulin (pmol/l) 4912	A	0.00 (−0.00; 0.01)	**–**	**–**	0.681
	B	−0.00 (− 0.01; 0.00)	–	–	
	C	−0.00 (− 0.01; 0.00)	–	–	
CCMR 4595 (boys: 2276; girls: 2319)	A	**0.014 (0.001; 0.028)**	**0.018 (0.002; 0.034)**	0.008 (− 0.017; 0.033)	0.030
	B	−0.00 (− 0.01; 0.01)	0.000 (− 0.020; 0.021)	− 0.003 (− 0.021; 0.014)	
CCMR_*no adip*_ 4595 (boys: 2276; girls: 2319)	A	0.02 (− 0.00; 0.03)	**0.022 (0.007; 0.037)**	0.007 (− 0023; 0.037)	0.036
	B	− 0.00 (− 0.01; 0.01)	0.002 (− 0.018; 0.022)	− 0.006 (− 0.028; 0.016)	
	C	0.00 (− 0.01; 0.01)	0.004 (− 0.011; 0.018)	− 0.005 (− 0.028; 0.017)	

Results are regression coefficients (95% CI) from meta-analysis, representing the difference in mean value of the outcome for every 1 h increase in PST. Statistically significant (P < 0.05) estimates are indicated in bold. Sex-specific associations are provided when a significant interaction with sex was found

Models A were adjusted for sex (in total group), age and wear time (h/day). Models B were additionally adjusted for time spent in moderate-to-vigorous physical activity. Models C were additionally adjusted for waist circumference

Abbrevations: *PST* prolonged sedentary time, *CCMR* clustered cardio-metabolic risk score including waist circumference, *CCMR*_*no adip*_ clustered cardio-metabolic risk score excluding waist circumference

### Isotemporal substitution of prolonged sedentary time and cardio-metabolic risk

Substitution of 1 h/day of PST > 15 min with the same duration of non-PST was estimated to reduce standardized BMI by 0.06 SD units (Table 4 and Additional file 1: Figure S1-S7). However, substitution with the same amount of MVPA was associated with a > 7-fold greater estimated reduction in standardized BMI (0.44 SD units). For waist circumference, systolic blood pressure, CCMR and CCMR_no adip_, only reallocation to MVPA was significantly associated with these outcomes. A 1 h/day substitution was associated with a 3.1 cm lower waist circumference (boys: 3.5 cm; girls: 2.7 cm), 1.5 mmHg lower systolic blood pressure (boys: 1.5 mmHg; girls: 1.9 mmHg) and a 0.18 SD unit lower CCMR (boys: 0.15 SD units; girls: 0.22 SD units). For HDL-cholesterol in boys (0.01 mmol/l) and diastolic blood pressure in the total group (− 0.2 mmHg) and girls (− 0.3 mmHg), substitution with LIPA suggested potential beneficial effects. However, substitution by MVPA resulted in stronger effect estimates, both for HDL-cholesterol (total group: 0.05 mmol/l; girls: 0.07 mmol/l; boys: 0.05 mmol/l) and diastolic blood pressure (total group: − 0.8 mmHg; girls: − 1.3 mmHg). Interestingly, substitution by non-PST was associated with higher levels of diastolic blood pressure (total group and boys: 0.3 mmHg; girls: 0.4 mmHg).

**Table 4:** Cross-sectional iso-temporal models examining substitution of prolonged sedentary time with non-prolonged sedentary time, light and moderate-to-vigorous physical activity, ICAD

Outcome		Substitution of PST > 15 min bouts (h/day)		
		With non-PST ≤ 15 min bouts (h/day)	With LIPA (h/day)	With MVPA (h/day)
Standardized BMI	**All**	**−0.06 (− 0.09; − 0.03)**	0.01 (− 0.02; 0.03)	**−0.44 (− 0.62; − 0.26)**
Waist circumference (cm)	**All**	−0.18 (− 0.46; 0.09)	−0.10 (− 0.32; 0.12)	**−3.07 (− 4.47; − 1.68)**
	**Boys**	−0.34 (− 0.70; 0.03)	−0.22 (− 0.49; 0.04)	**−3.46 (− 4.92; − 2.00)**
	**Girls**	−0.06 (− 0.44; 0.31)	0.02 (− 0.24; 0.27)	**− 2.65 (− 4.10; − 1.19)**
HDL-cholesterol (mmol/l)	**All**	0.000 (− 0.018; 0.019)	0.000 (− 0.006; 0.006)	**0.054 (0.005; 0.104)**
	**Boys**	0.003 (− 0.015; 0.020)	**0.010 (0.001; 0.019)**	0.048 (− 0.013; 0.108)
	**Girls**	− 0.002 (− 0.026; 0.023)	−0.007 (− 0.017; 0.004)	**0.073 (0.034; 0.111)**
Systolic blood pressure (mmHg)	**All**	−0.03 (− 0.33; 0.27)	−0.04 (− 0.22; 0.14)	**−1.53 (− 2.42; − 0.65)**
	**Boys**	0.01 (− 0.35; 0.36)	−0.03 (− 0.24; 0.17)	**−1.48 (− 2.54; − 0.43)**
	**Girls**	−0.15 (− 0.54; 0.24)	−0.05 (− 0.42; 0.32)	**−1.91 (− 3.09; − 0.73)**
Diastolic blood pressure (mmHg)	**All**	**0.33 (0.06; 0.60)**	**−0.16 (− 0.28; − 0.04)**	**−0.81 (− 1.38; − 0.24)**
	**Boys**	**0.31 (0.03; 0.60)**	− 0.04 (− 0.20; 0.13)	−0.55 (− 1.34; 0.24)
	**Girls**	**0.41 (0.01; 0.82)**	**−0.28 (− 0.48; − 0.07)**	**−1.33 (− 2.14; − 0.51)**
CCMR	**All**	0.01 (− 0.01; 0.03)	−0.00 (− 0.02; 0.01)	**−0.18 (− 0.27; − 0.10)**
	**Boys**	0.02 (− 0.01; 0.05)	−0.01 (− 0.03; 0.02)	**−0.15 (− 0.23; − 0.06)**
	**Girls**	−0.01 (− 0.04; 0.02)	0.01 (− 0.01; 0.04)	**−0.22 (− 0.33; − 0.11)**
CCMR_*no adip*_	**All**	0.01 (− 0.01; 0.04)	−0.00 (− 0.02; 0.01)	**−0.20 (− 0.29; − 0.11)**
	**Boys**	0.03 (− 0.01; 0.07)	−0.01 (− 0.03; 0.02)	**−0.15 (− 0.25; − 0.06)**
	**Girls**	−0.01 (− 0.04; 0.02)	0.01 (− 0.02; 0.04)	**−0.24 (− 0.36; − 0.12)**

Results are regression coefficients (95% CI) from meta-analysis, representing the difference in the outcome variable when increasing that type of activity by 1 h/day while decreasing PST by the same duration and holding other activity components constant. Differences in outcome variables when modelling 30 min/day substitutions instead of 1 h/day substitutions (holding other activity components constant) equate to 50% of the estimates presented above. Statistically significant (*P* < 0.05) estimates are indicated in bold. Sex-specific associations are provided when a significant interaction with sex was found in Table 3

Models omitted the PST variable under study and incorporated the complementary non-prolonged ST variable of interest, LIPA and MVPA, and are adjusted for wear time (h/day), sex (in total group) and age

Abbrevations: *PST* prolonged sedentary time, *non-PST* non-prolonged sedentary time, *LIPA* light-intensity physical activity, *MVPA* moderate-to-vigorous physical activity, *CCMR* clustered cardio-metabolic risk score including waist circumference, *CCMR*_*no adip*_ clustered cardio-metabolic risk score excluding waist circumference

N included by outcome: see Table 3

## Discussion

Constituting almost a third of all sedentary time and a substantial proportion (16%) of daily waking wear time in this age group, PST > 15 min is a potentially important target for behaviour change. Modelling 1 h/day substitutions of PST with higher intensity activity suggested beneficial effects on most cardio-metabolic health outcomes examined; however, a moderate-to-vigorous intensity of replacement activity seemed preferable. Replacement with LIPA was only associated beneficially with HDL cholesterol (boys) and diastolic blood pressure. Similarly, findings suggested that replacement with more interrupted sedentary time may result in limited benefit, based on weak associations with only BMI and diastolic blood pressure (the latter in the unexpected direction).

Effect estimates for replacement with MVPA are clinically relevant. For example, the estimated reduction found for waist circumference of ≈3 cm would be associated with an approximately 6% lower risk of cardiovascular events [31]. A 1 h/day replacement with MVPA may however be difficult to achieve, as it is equivalent to an increase of 200% of mean observed MVPA in this sample, compared to a potentially more feasible increase by 16 and 24% of mean observed LIPA and non-PST, respectively. However, when modelling 30 min/day replacements with MVPA, the estimated reductions in waist circumference and subsequent incident CVD risk would be ≈1.5 cm and 3%, respectively, which are still clinically important [31]. Compared to a recent cross-sectional study examining substitution of total, rather than prolonged sedentary time, by MVPA, we found stronger effect estimates for systolic blood pressure and HDL-cholesterol, albeit comparable effect estimates for waist circumference [32]. This supports the notion that focusing on replacements of prolonged sedentary time may be a more efficient way to preserve cardio-metabolic health in children, rather than focusing on all sedentary time.

Observational studies in children on accumulation patterns in sedentary time and cardio-metabolic health have focused on independent rather than iso-temporal associations. Differences in exposure definition, accelerometry methodology, analysis strategy and to some extent study population between our meta-analysis and other singular studies complicate comparisons of results [3–5]. However, attenuation of most associations following adjustment for MVPA is in line with the literature [3, 4]. Sex-specific associations were similarly found in some studies that suggested somewhat stronger associations in boys [33, 34], although this was not corroborated by others [35, 36]. Although true biological differences could explain our findings, a more plausible explanation might be found in the type of prolonged sitting. For example, boys engage in more screen time, which is more strongly associated with impaired cardiovascular health [37, 38]. This could be mediated through dietary alterations, or the interplay with the timing of energy-dense meal consumption [38]. Prolonged sitting following energy-dense meals has been shown to exacerbate post-prandial glucose and lipid excursions [39, 40]. Future work should examine whether PST at specific times of the day is more strongly associated with cardio-metabolic health, and hence whether it would be more effective, but also feasible, to replace PST by other activities during these time periods. Insights into mechanistic pathways between prolonged sitting and cardio-metabolic health are still limited, need further scrutiny and are predominantly based on animal and adult human populations. However, hypothesized mechanisms include reductions in muscular demand, blood flow, lipid oxidation, muscle/liver insulin sensitivity and vascular function, and increased body insulin resistance, ectopic fat storage, and oxidative stress [41]. Finally, even though our findings suggest most beneficial effects for replacement with MVPA, the optimal accumulation patterns need further investigation, including MVPA bout durations and activity type (aerobic versus resistance). This may well differ depending on the outcome (e.g. glucose or lipid metabolism, vascular function) [39, 42].

Important strengths of this study include the large sample size and wide age range, allowing for investigation of moderation effects by sex and age, which is more challenging in smaller cohort and intervention studies. The great heterogeneity of included study samples in terms of ethnicity, obesity status and general health profile also increases external validity of the findings. Second, meta-analysis rather than pooled analysis allowed for better control of residual confounding through sensitivity analyses allowing differential level of adjustment between studies. Third, harmonization of all exposure variables further increased robustness of the results and allowed for estimation of non-iso-temporal and iso-temporal effect estimates, which is novel in children and youth, especially on this scale. All exposures were also measured by accelerometry, rather than by self-report, avoiding biases associated with the latter. Finally, our characterization of prolonged sedentary time based on time spent in sedentary bouts of minimal duration is more optimal compared to other methods, such as breaks in sedentary time [6, 29].

The following limitations however also need to be considered. The observational and cross-sectional study design prevents conclusions in terms of causality and effect estimates are based on statistical modeling rather than behavioural change. It is, however, unlikely that children with less healthy profiles for non-adiposity risk factors (mostly subclinical in this age group) would have consequentially changed their sedentary time accumulation habits. The alternative direction of causality, i.e. prolonged sitting increasing cardio-metabolic risk, is in line with findings from intervention studies in children, showing acute beneficial effects of interrupting sitting time by activity breaks on glucose, lipid and vascular function [39, 42]. The accelerometry used in the included studies does not distinguish between postures, which may have resulted in a certain degree of misclassification between standing and non-standing posture. Participants on average wore the accelerometers for 5 days, which may not fully reflect their habitual activity levels due to high within-individual variability. As previously estimated, this may have led to an underestimation of the true magnitude of associations by 50% [1]. Sensitivity analyses excluding those without valid measurement on a weekend day and at least three valid days did not alter results. The latter, in combination with high wear time adherence during awake time (i.e. mean wear time of 13 h/day across an average of 5 days) also suggests that our findings are robust to alternative valid day definitions. Although our intensity threshold for MVPA could be considered fairly high, implementing a threshold of 2000 cpm did not materially change results (data not shown). Finally, due to data unavailability we could not include dietary intake as a covariate in our analyses. Hence residual confounding may be at play, also for variables which were not available in all studies and were hence only included for some studies in our sensitivity analyses. Future studies should aim to examine the extent of this issue more comprehensively.

## Conclusions

Our findings suggest that replacements of PST with higher-intensity activity, preferably MVPA, are beneficially associated with adiposity, HDL-cholesterol, blood pressure and clustered cardio-metabolic risk in children and adolescents, with clinically relevant effect estimates. Future observational studies, with accelerometry and cardio-metabolic profiling at multiple time-points, should examine whether these iso-temporal associations persist in longitudinal analysis, to gain further insights in these associations in the long term according to important population strata such as age and sex. These should be accompanied by further lab-based and free-living intervention studies, respectively examining the acute and more chronic effects of such substitutions on cardio-metabolic health in children.

## Supplementary information

**Additional file 1: Table S1.** Individual cohort characteristics and descriptive statistics of cardio-metabolic outcome variables, ICAD. **Table S2.** Cross-sectional associations between prolonged sedentary time, defined as > 30 min bouts, and cardio-metabolic risk, ICAD. **Table S3.** Cross-sectional associations between prolonged sedentary time and cardio-metabolic risk, ICAD (> = 3 valid day with > = 1 valid weekend day). **Table S4.** Cross-sectional associations between prolonged sedentary time and cardio-metabolic risk with more elaborate adjustment for confounding, ICAD (> = 1 valid day). **Figure S1.** Meta-analysis for standardised BMI, showing regression coefficients (95% CI) from cross-sectional iso-temporal models examining substitution of prolonged sedentary time with non-prolonged sedentary time, light and moderate-to-vigorous physical activity, ICAD. **Figure S2.** Meta-analysis for waist circumference, showing regression coefficients (95% CI) from cross-sectional iso-temporal models examining substitution of prolonged sedentary time with non-prolonged sedentary time, light and moderate-to-vigorous physical activity, ICAD. **Figure S3.** Meta-analysis for HDL cholesterol, showing regression coefficients (95% CI) from cross-sectional iso-temporal models examining substitution of prolonged sedentary time with non-prolonged sedentary time, light and moderate-to-vigorous physical activity, ICAD. **Figure S4.** Meta-analysis for systolic blood pressure, showing regression coefficients (95% CI) from cross-sectional iso-temporal models examining substitution of prolonged sedentary time with non-prolonged sedentary time, light and moderate-to-vigorous physical activity, ICAD. **Figure S5.** Meta-analysis for diastolic blood pressure, showing regression coefficients (95% CI) from cross-sectional iso-temporal models examining substitution of prolonged sedentary time with non-prolonged sedentary time, light and moderate-to-vigorous physical activity, ICAD. **Figure S6.** Meta-analysis for clustered cardio-metabolic risk, showing regression coefficients (95% CI) from cross-sectional iso-temporal models examining substitution of prolonged sedentary time with non-prolonged sedentary time, light and moderate-to-vigorous physical activity, ICAD. **Figure S7.** Meta-analysis for clustered cardio-metabolic risk excluding waist circumference, showing regression coefficients (95% CI) from cross-sectional iso-temporal models examining substitution of prolonged sedentary time with non-prolonged sedentary time, light and moderate-to-vigorous physical activity, ICAD (DOCX 173 kb)

## Data Availability

The datasets supporting the conclusions of this article are available via http://www.mrc-epid.cam.ac.uk/Research/Studies/ICAD/.

## Abbreviations

ALSPAC: Avon Longitudinal Study of Parents and Children
BMI: Body mass index
BP: Blood pressure
CCMR: Clustered cardio-metabolic risk
CCMR_no adip_: Clustered cardio-metabolic risk without central adiposity component
cpm: Counts per minute
CSCIS: Copenhagen School Child Intervention Study
EYHS: European Youth Heart Study
ICAD: International Children’s Accelerometry Database
IQR: Interquartile range
KISS: Kinder Sportstudie
LIPA: Light-intensity physical activity
MAGIC: Movement and Activity Glasgow Intervention in Children
MVPA: Moderate-to-vigorous physical activity
NHANES: National Health and Nutrition Examination Survey
non-PST: Non-prolonged sedentary time
PEACH: Personal and Environmental Associations with Children’s Health
PST: Prolonged sedentary time
SD: Standard deviation
SES: Socio-economic status
SPEEDY: Sport, Physical Activity and Eating Behavior: Environmental Determinants in Young People
Triglyc: Triglycerides
Waist: Waist circumference
