# Supplementary material for: Substituting prolonged sedentary time and cardiovascular risk in children and youth: a meta-analysis within the International Children’s Accelerometry database (ICAD)
Source: Int J Behav Nutr Phys Act. 2019 Oct 31;16:96. doi: 10.1186/s12966-019-0858-6 (PMC6822444; doi:10.1186/s12966-019-0858-6)
Supplement: Supplementary file 1 — Additional file 1: Table S1. Individual cohort characteristics and descriptive statistics of cardio-metabolic outcome variables, ICAD. Table S2. Cross-sectional associations between prolonged sedentary time, defined as > 30 min bouts, and cardio-metabolic risk, ICAD. Table S3. Cross-sectional associations between prolonged sedentary time and cardio-metabolic risk, ICAD (> = 3 valid day with > = 1 valid weekend day). Table S4. Cross-sectional associations between prolonged sedentary time and cardio-metabolic risk with more elaborate adjustment for confounding, ICAD (> = 1 valid day). Figure S1. Meta-analysis for standardised BMI, showing regression coefficients (95% CI) from cross-sectional iso-temporal models examining substitution of prolonged sedentary time with non-prolonged sedentary time, light and moderate-to-vigorous physical activity, ICAD. Figure S2. Meta-analysis for waist circumference, showing regression coefficients (95% CI) from cross-sectional iso-temporal models examining substitution of prolonged sedentary time with non-prolonged sedentary time, light and moderate-to-vigorous physical activity, ICAD. Figure S3. Meta-analysis for HDL cholesterol, showing regression coefficients (95% CI) from cross-sectional iso-temporal models examining substitution of prolonged sedentary time with non-prolonged sedentary time, light and moderate-to-vigorous physical activity, ICAD. Figure S4. Meta-analysis for systolic blood pressure, showing regression coefficients (95% CI) from cross-sectional iso-temporal models examining substitution of prolonged sedentary time with non-prolonged sedentary time, light and moderate-to-vigorous physical activity, ICAD. Figure S5. Meta-analysis for diastolic blood pressure, showing regression coefficients (95% CI) from cross-sectional iso-temporal models examining substitution of prolonged sedentary time with non-prolonged sedentary time, light and moderate-to-vigorous physical activity, ICAD. Figure S6. Meta-analysis for clustered [file 12966_2019_858_MOESM1_ESM.docx]

**Additional Table 1.** Individual cohort characteristics and descriptive statistics of cardio-metabolic outcome variables, *ICAD*

| **Study** | **Country, measurement year** | **N participants** | | **Age range,**  **years** | **Standar-dised**  **BMI** | **Waist,**  **cm** | **Triglyc†, mmol/l** | **HDL, mmol/l** | **Systolic BP,**  **mmHg** | **Diastolic BP,**  **mmHg** | **Fasting plasma glucose, mmol/l** | **Fasting**  **serum**  **insulin†,**  **pmol/l** |
| --- | --- | --- | --- | --- | --- | --- | --- | --- | --- | --- | --- | --- |
|  |  | **Boys** | **Girls** |  |  |  |  |  |  |  |  |  |
| **ALSPAC** | United Kingdom,  2003-2005 | 2,841 | 3109 | 10.6 – 13.6 | 0.3 (1.2) | 68.2 (9.4) | - | - | 105.5 (9.9) | 58.7 (6.5) | - | - |
| **Ballabeina** | Switzerland,  2008 | 181 | 183 | 3.9 – 6.4 | 0.1 (1.0) | 52.9 (4.2) | - | - | - | - | - | - |
| **CSCIS** | Denmark,  2001-2003 | 318 | 277 | 5.8 – 8.2 | 0.1 (1.0) | 55.6 (5.3) | 0.5 (0.4 – 0.7) | 1.5 (0.3) | 97.9 (8.0) | 57.7 (5.8) | 4.3 (0.4) | 24.6 (16.6 – 34.9) |
| **EYHS Denmark** | Denmark,  1997-1998; 2003-2004 | 549 | 683 | 8.4 – 18.4 | 0.2 (1.0) | 62.5 (7.7) | 0.8 (0.6 – 1.0) | 1.5 (0.3) | 104.9 (10.2) | 61.5 (6.3) | 5.1 (0.4) | 48.3 (33.6 – 70.1) |
| **EYHS Estonia** | Estonia,  1998-1999 | 293 | 366 | 8.3 – 17.1 | 0.0 (1.0) | 62.4 (8.1) | 0.7 (0.6 – 0.9) | 1.4 (0.3) | 105.9 (11.3) | 61.2 (7.1) | 5.1 (0.4) | 49.6 (32.9 – 69.4) |
| **EYHS Norway** | Norway,  1999-2000 | 194 | 190 | 8.9 – 10.4 | 0.1 (1.0) | 60.3 (5.7) | 0.8 (0.7 – 0.9) | 1.5 (0.3) | 102.9 (7.6) | 62.6 (5.8) | - | - |
| **EYHS Portugal** | Portugal,  1999-2000 | 599 | 591 | 8.2 – 16.4 | 0.6 (1.2) | 63.3 (8.2) | 0.6 (0.5 – 0.9) | 1.6 (0.3) | 97.0 (9.8) | 55.0 (6.4) | 5.2 (0.4) | 30.6 (18.0 – 45.9) |
| **KISS** | Switzerland,  2005 | 192 | 208 | 6.0 – 13.0 | 0.2 (1.1) | 58.5 (7.2) | 0.6 (0.4 – 0.7) | 1.6 (0.3) | - | - | 4.6 (0.4) | 39.0 (27.6 – 54.6) |
| **MAGIC** | United Kingdom,  2002 | 205 | 214 | 3.6 – 4.9 | 0.4 (1.0) | 51.3 (3.6) | - | - | 97.1 (7.8) | 60.8 (6.5) | - | - |
| **NHANES 2003-2004** | United States,  2003-2004 | 1,167 | 1,108 | 6.0 – 18.0 | 0.9 (1.3) | 74.8 (15.5) | 0.8 (0.6 – 1.1) | 1.4 (0.3) | 106.7 (10.3) | 57.5 (11.6) | 5.0 (0.5) | 55.4 (36.8 – 89.6) |
| **NHANES 2005-2006** | United States,  2005-2006 | 1,186 | 1,193 | 6.0 – 18.0 | 0.8 (1.4) | 74.4 (16.0) | 0.8 (0.6 – 1.1) | 1.4 (0.3) | 107.7 (10.4) | 58.0 (10.9) | 5.2 (0.4) | 61.3 (41.2 – 93.6) |
| **PEACH** | United Kingdom,  2006-2008 | 596 | 624 | 10.0 – 11.9 | 0.4 (1.2) | 66.9 (9.1) | - | - | - | - | - | - |
| **Pelotas** | Brazil,  2006-2007 | 238 | 218 | 12.7 – 14.0 | 0.4 (1.2) | 68.6 (8.4) | - | - | 110.6 (14.1) | 68.2 (11.2) | - | - |
| **SPEEDY** | United Kingdom,  2007 | 881 | 1,098 | 9.5 – 11.4 | 0.4 (1.2) | 64.1 (8.4) | - | - | - | - | - | - |

Cardio-metabolic outcome variables are presented as means (SD), except those indicated with † which are presented as medians (IQR) due to a skewed distribution. N for variables other than waist circumference may be lower, due to missing data (n across studies for each variable is indicated in Table 3).

Abbreviations: ALSPAC: Avon Longitudinal Study of Parents and Children; CSCIS: Copenhagen School Child Intervention Study; EYHS: European Youth Heart Study; KISS: Kinder Sportstudie; MAGIC: Movement and Activity Glasgow Intervention in Children; NHANES: National Health and Nutrition Examination Survey; PEACH: Personal and Environmental Associations with Children’s Health; SPEEDY: Sport, Physical Activity and Eating Behavior: Environmental Determinants in Young People; Waist: waist circumference; Triglyc: triglycerides; BP: blood pressure.

**Additional Table 2.** Cross-sectional associations between prolonged sedentary time, defined as >30 min bouts, and cardio-metabolic risk, ICAD

| **Outcome/**  N included | **Model** | **PST, >30 min bouts (h/day)** | | |
| --- | --- | --- | --- | --- |
|  |  | **All** | **Boys** | **Girls** |
| **Standardized BMI**  19,477 | A | **0.033 (0.003; 0.063)** | **-** | **-** |
|  | B | 0.01 (-0.02; 0.03) | - | - |
| **Waist circumference (cm)**  19,502  (boys: 9,440; girls: 10,062) | A | 0.24 (-0.04; 0.52) | **0.40 (0.04; 0.76)** | 0.10 (-0.22; 0.42) |
|  | B | 0.05 (-0.19; 0.29) | 0.13 (-0.17; 0.43) | -0.02 (-0.32; 0.27) |
| **Triacylglycerol (mmol/l)**  5,330 | A | 0.001 (-0.001; 0.004) | **-** | **-** |
|  | B | -0.001 (-0.003; 0.002) | - | - |
|  | C | -0.001 (-0.003; 0.002) | - | - |
| **HDL-cholesterol (mmol/l)**  7,849  (boys: 3,918; girls: 4,031) | A | -0.01 (-0.02; 0.01) | **-0.014 (-0.025; -0.003)** | 0.001 (-0.010; 0.012) |
|  | B | -0.001 (-0.013; 0.010) | -0.010 (-0.024; 0.005) | 0.005 (-0.005; 0.015) |
|  | C | -0.003 (-0.012; 0.006) | -0.010 (-0.022; 0.002) | 0.002 (-0.007; 0.012) |
| **Systolic blood pressure (mmHg)**  14,726  (boys: 7,177; girls: 7,549) | A | 0.14 (-0.03; 0.31) | 0.10 (-0.14; 0.34) | 0.24 (-0.02; 0.50) |
|  | B | 0.01 (-0.17; 0.18) | -0.02 (-0.27; 0.23) | 0.08 (-0.16; 0.32) |
|  | C | -0.01 (-0.18; 0.15) | -0.05 (-0.28; 0.19) | 0.06 (-0.17; 0.30) |
| **Diastolic blood pressure (mmHg)**  14,687  (boys: 7,155; girls: 7,532) | A | 0.10 (-0.03; 0.23) | -0.02 (-0.22; 0.17) | **0.20 (0.02; 0.39)** |
|  | B | -0.01 (-0.15; 0.13) | -0.11 (-0.31; 0.09) | 0.05 (-0.14; 0.24) |
|  | C | -0.05 (-0.19; 0.08) | -0.17 (-0.37; 0.03) | 0.01 (-0.17; 0.20) |
| **Fasting plasma glucose (mmol/l)**  4,952 | A | 0.007 (-0.004; 0.018) | **-** | **-** |
|  | B | 0.00 (-0.01; 0.01) | - | - |
|  | C | 0.00 (-0.01; 0.01) | - | - |
| **Fasting serum insulin (pmol/l)**  4,912 | A | 0.00 (-0.01; 0.01) | **-** | **-** |
|  | B | -0.007 (-0.016; 0.002) | - | - |
|  | C | -0.005 (-0.013; 0.004) | - | - |
| **CCMR**  4,595  (boys: 2,276; girls: 2,319) | A | 0.013 (-0.005; 0.031) | **-** | **-** |
|  | B | -0.002 (-0.020; 0.015) | - | - |
| **CCMR*_no adip_***  4,595  (boys: 2,276; girls: 2,319) | A | 0.015 (-0.005; 0.035) | **-** | **-** |
|  | B | -0.001 (-0.019; 0.016) | - | - |
|  | C | 0.003 (-0.010; 0.016) | - | - |

Results are regression coefficients (95% CI) from meta-analysis, representing the difference in mean value of the outcome for every 1 hour increase in PST. Statistically significant (P <0.05) estimates are indicated in bold. Sex-specific associations are provided when a significant interaction with sex was found.

Models A were adjusted for sex (in total group), age and wear time (h/day). Models B were additionally adjusted for time spent in moderate-to-vigorous physical activity. Models C were additionally adjusted for waist circumference.

Abbrevations: PST: prolonged sedentary time; CCMR: clustered cardio-metabolic risk score including waist circumference; CCMR*_no adip_*: clustered cardio-metabolic risk score excluding waist circumference.

**Additional Table 3.** Cross-sectional associations between prolonged sedentary time and cardio-metabolic risk, ICAD (>=3 valid day with >=1 valid weekend day)

| **Outcome** | **n** | **Model** | **PST, >15 min bouts**  **(h/day)** | **PST, >30 min bouts**  **(h/day)** |
| --- | --- | --- | --- | --- |
| **Standardized BMI** | 15,982 | A | **0.04 (0.00; 0.07)** | **0.05 (0.01; 0.09)** |
|  |  | B | 0.01 (-0.01; 0.03 ) | 0.02 (-0.01; 0.05) |
| **Waist circumference**  **(cm)** | 16,004 | A | **0.37 (0.06; 0.68)** | **0.39 (0.02; 0.76)** |
|  |  | B | 0.14 (-0.10; 0.38) | 0.15 (-0.15; 0.45) |
| **Triacylglycerol**  **(mmol/l)** | 4,262 | A | **0.003 (0.001; 0.005)** | **0.003 (0.000; 0.005)** |
|  |  | B | 0.001 (-0.001; 0.004) | 0.001 (-0.002; 0.004) |
|  |  | C | 0.001 (-0.001; 0.003) | 0.001 (-0.002; 0.003) |
| **HDL-cholesterol**  **(mmol/l)** | 6,293 | A | -0.01 (-0.02; 0.00) | -0.01 (-0.02; 0.01) |
|  |  | B | -0.002 (-0.009; 0.005) | -0.00 (-0.02; 0.01) |
|  |  | C | -0.00 (-0.01; 0.01) | -0.00 (-0.02; 0.01) |
| **Systolic blood pressure**  **(mmHg)** | 12,083 | A | **0.31 (0.15; 0.46)** | **0.37 (0.16; 0.59)** |
|  |  | B | 0.16 (-0.01; 0.32) | 0.22 (-0.00; 0.45) |
|  |  | C | 0.08 (-0.08; 0.23) | 0.14 (-0.07; 0.35) |
| **Diastolic blood pressure**  **(mmHg)** | 12,054 | A | **0.23 (0.11; 0.35)** | 0.17 (-0.00; 0.33) |
|  |  | B | **0.14 (0.01; 0.26)** | 0.05 (-0.12; 0.23) |
|  |  | C | 0.09 (-0.03; 0.22) | 0.00 (-0.17; 0.17) |
| **Fasting plasma glucose**  **(mmol/l)** | 3,951 | A | 0.01 (-0.00; 0.02) | **0.02 (0.00; 0.03)** |
|  |  | B | 0.00 (-0.01; 0.01) | 0.01 (-0.01; 0.02) |
|  |  | C | 0.00 (-0.01; 0.01) | 0.01 (-0.01; 0.02) |
| **Fasting serum insulin**  **(pmol/l)** | 3,919 | A | 0.00 (-0.00; 0.01) | 0.00 (-0.00; 0.01) |
|  |  | B | -0.005 (-0.012; 0.001) | -0.006 (-0.014; 0.002) |
|  |  | C | -0.005 (-0.012; 0.001) | -0.006 (-0.013; 0.002) |
| **CCMR** | 3,637 | A | **0.02 (0.01; 0.04)** | **0.03 (0.01; 0.05)** |
|  |  | B | 0.01 (-0.01; 0.02) | 0.01 (-0.01; 0.03) |
| **CCMR*_no adip_*** | 3,637 | A | **0.03 (0.01; 0.04)** | **0.03 (0.02; 0.05)** |
|  |  | B | 0.01 (-0.01; 0.02) | 0.01 (-0.00; 0.03) |
|  |  | C | 0.01 (-0.01; 0.02) | 0.01 (-0.01; 0.03) |

Results are regression coefficients (95% CI) from meta-analysis, representing the difference in mean value of the outcome for every 1 hour increase in PST. Statistically significant (P <0.05) estimates are indicated in bold.

Models A were adjusted for sex, age and wear time (h/day). Models B were additionally adjusted for time spent in moderate-to-vigorous physical activity. Models C were additionally adjusted for waist circumference.

Abbrevations: PST: prolonged sedentary time; CCMR: clustered cardio-metabolic risk score including waist circumference; CCMR*_no adip_*: clustered cardio-metabolic risk score excluding waist circumference.

**Additional Table 4.** Cross-sectional associations between prolonged sedentary time and cardio-metabolic risk with more elaborate adjustment for confounding, ICAD (>=1 valid day)

| **Outcome** | **n** | **Model** | **PST, >15 min bouts**  **(h/day)** | **PST, >30 min bouts**  **(h/day)** |
| --- | --- | --- | --- | --- |
| **Standardized BMI** | 19,477 | A | 0.02 (-0.01; 0.04) | 0.01 (-0.02; 0.05) |
|  |  | B | -0.00 (-0.03; 0.02) | -0.01 (-0.04; 0.02) |
| **Waist circumference**  **(cm)** | 19,502 | A | 0.21 (-0.02; 0.45) | 0.15 (-0.13; 0.42) |
|  |  | B | 0.05 (-0.16; 0.26) | -0.02 (-0.28; 0.24) |
| **Triacylglycerol**  **(mmol/l)** | 5,330 | A | 0.000 (-0.002; 0.003) | 0.000 (-0.002; 0.003) |
|  |  | B | -0.001 (-0.004; 0.003) | -0.001 (-0.005; 0.002) |
|  |  | C | -0.000 (-0.003; 0.003) | -0.000 (-0.004; 0.003) |
| **HDL-cholesterol**  **(mmol/l)** | 7,849 | A | -0.003 (-0.009; 0.003) | -0.00 (-0.01; 0.01) |
|  |  | B | 0.002 (-0.004; 0.008) | 0.00 (-0.01; 0.01) |
|  |  | C | -0.000 (-0.006; 0.006) | 0.00 (-0.01; 0.01) |
| **Systolic blood pressure**  **(mmHg)** | 14,726 | A | 0.07 (-0.07; 0.22) | 0.03 (-0.16; 0.23) |
|  |  | B | -0.06 (-0.22; 0.09) | -0.10 (-0.30; 0.10) |
|  |  | C | -0.06 (-0.21; 0.09) | -0.06 (-0.25; 0.13) |
| **Diastolic blood pressure**  **(mmHg)** | 14,687 | A | **0.16 (0.04; 0.28)** | 0.08 (-0.09; 0.24) |
|  |  | B | 0.08 (-0.05; 0.20) | -0.02 (-0.19; 0.15) |
|  |  | C | 0.05 (-0.08; 0.18) | -0.06 (-0.22; 0.11) |
| **Fasting plasma glucose**  **(mmol/l)** | 4,952 | A | 0.00 (-0.01; 0.01) | 0.00 (-0.01; 0.01) |
|  |  | B | -0.01 (-0.02; 0.00) | -0.00 (-0.02; 0.01) |
|  |  | C | -0.01 (-0.02; 0.00) | -0.00 (-0.02; 0.01) |
| **Fasting serum insulin**  **(pmol/l)** | 4,912 | A | 0.001 (-0.005; 0.006) | -0.003 (-0.010; 0.004) |
|  |  | B | -0.006 (-0.011; 0.000) | **-0.01 (-0.02; -0.00)** |
|  |  | C | -0.004 (-0.009; 0.001) | -0.01 (-0.01; 0.00) |
| **CCMR** | 4,595 | A | **0.012 (0.00; 0.023)** | 0.01 (-0.01; 0.02) |
|  |  | B | -0.002 (-0.014; 0.010) | -0.01 (-0.02; 0.01) |
| **CCMR*_no adip_*** | 4,595 | A | 0.01 (0.00; 0.03) | 0.01 (-0.01; 0.03) |
|  |  | B | -0.00 (-0.01; 0.01) | -0.01 (-0.02; 0.01) |
|  |  | C | 0.00 (-0.01; 0.01) | -0.00 (-0.02; 0.01) |

Results are regression coefficients (95% CI) from meta-analysis, representing the difference in mean value of the outcome for every 1 hour increase in PST. Statistically significant (P <0.05) estimates are indicated in bold.

Models A were adjusted for sex, age and wear time (h/day) in all studies. Additional adjustment was made for ethnicity (10 studies), parental SES (8 studies), birth weight (8 studies) and sexual maturity (6 studies). Models B were additionally adjusted for time spent in moderate-to-vigorous physical activity. Models C were additionally adjusted for waist circumference.

Abbrevations: PST: prolonged sedentary time; CCMR: clustered cardio-metabolic risk score including waist circumference; CCMR*_no adip_*: clustered cardio-metabolic risk score excluding waist circumference.

**Additional Figure 1.** Meta-analysis for standardised BMI, showing regression coefficients (95% CI) from cross-sectional iso-temporal models examining substitution of prolonged sedentary time with non-prolonged sedentary time, light and moderate-to-vigorous physical activity, ICAD

Additional Figure 1 legend:

Regression coefficients (95% CI) represent the difference in standardised BMI when increasing that type of activity by 1 h/day while decreasing prolonged sedentary time by the same duration and holding other activity components constant. Differences in outcome variables when modelling 30 min/day substitutions instead of 1h/day substitutions (holding other activity components constant) equate to 50% of the estimates presented above. Models omitted prolonged sedentary time and incorporated non-prolonged sedentary time, light and moderate-to-vigorous physical activity and are adjusted for wear time (h/day), sex and age.

**Additional Figure 2.** Meta-analysis for waist circumference, showing regression coefficients (95% CI) from cross-sectional iso-temporal models examining substitution of prolonged sedentary time with non-prolonged sedentary time, light and moderate-to-vigorous physical activity, ICAD

Additional Figure 2 legend:

Regression coefficients (95% CI) represent the difference in waist circumference (cm) when increasing that type of activity by 1 h/day while decreasing prolonged sedentary time by the same duration and holding other activity components constant. Differences in waist circumference when modelling 30 min/day substitutions instead of 1h/day substitutions (holding other activity components constant) equate to 50% of the estimates presented above. Models omitted prolonged sedentary time and incorporated non-prolonged sedentary time, light and moderate-to-vigorous physical activity and are adjusted for wear time (h/day), sex and age.

**Additional Figure 3.** Meta-analysis for HDL cholesterol, showing regression coefficients (95% CI) from cross-sectional iso-temporal models examining substitution of prolonged sedentary time with non-prolonged sedentary time, light and moderate-to-vigorous physical activity, ICAD

Additional Figure 3 legend:

Regression coefficients (95% CI) represent the difference in HDL cholesterol (mmol/l) when increasing that type of activity by 1 h/day while decreasing prolonged sedentary time by the same duration and holding other activity components constant. Differences in HDL cholesterol when modelling 30 min/day substitutions instead of 1h/day substitutions (holding other activity components constant) equate to 50% of the estimates presented above. Models omitted prolonged sedentary time and incorporated non-prolonged sedentary time, light and moderate-to-vigorous physical activity and are adjusted for wear time (h/day), sex and age.

**Additional Figure 4.** Meta-analysis for systolic blood pressure, showing regression coefficients (95% CI) from cross-sectional iso-temporal models examining substitution of prolonged sedentary time with non-prolonged sedentary time, light and moderate-to-vigorous physical activity, ICAD

Additional Figure 4 legend:

Regression coefficients (95% CI) represent the difference in systolic blood pressure (mmHg) when increasing that type of activity by 1 h/day while decreasing prolonged sedentary time by the same duration and holding other activity components constant. Differences in systolic blood pressure when modelling 30 min/day substitutions instead of 1h/day substitutions (holding other activity components constant) equate to 50% of the estimates presented above. Models omitted prolonged sedentary time and incorporated non-prolonged sedentary time, light and moderate-to-vigorous physical activity and are adjusted for wear time (h/day), sex and age.

**Additional Figure 5.** Meta-analysis for diastolic blood pressure, showing regression coefficients (95% CI) from cross-sectional iso-temporal models examining substitution of prolonged sedentary time with non-prolonged sedentary time, light and moderate-to-vigorous physical activity, ICAD

Additional Figure 5 legend:

Regression coefficients (95% CI) represent the difference in diastolic blood pressure (mmHg) when increasing that type of activity by 1 h/day while decreasing prolonged sedentary time by the same duration and holding other activity components constant. Differences in diastolic blood pressure when modelling 30 min/day substitutions instead of 1h/day substitutions (holding other activity components constant) equate to 50% of the estimates presented above. Models omitted prolonged sedentary time and incorporated non-prolonged sedentary time, light and moderate-to-vigorous physical activity and are adjusted for wear time (h/day), sex and age.

**Additional Figure 6.** Meta-analysis for clustered cardio-metabolic risk, showing regression coefficients (95% CI) from cross-sectional iso-temporal models examining substitution of prolonged sedentary time with non-prolonged sedentary time, light and moderate-to-vigorous physical activity, ICAD

Additional Figure 6 legend:

Regression coefficients (95% CI) represent the difference in clustered cardio-metabolic risk when increasing that type of activity by 1 h/day while decreasing prolonged sedentary time by the same duration and holding other activity components constant. Differences in clustered cardio-metabolic risk when modelling 30 min/day substitutions instead of 1h/day substitutions (holding other activity components constant) equate to 50% of the estimates presented above. Models omitted prolonged sedentary time and incorporated non-prolonged sedentary time, light and moderate-to-vigorous physical activity and are adjusted for wear time (h/day), sex and age.

**Additional Figure 7.** Meta-analysis for clustered cardio-metabolic risk excluding waist circumference, showing regression coefficients (95% CI) from cross-sectional iso-temporal models examining substitution of prolonged sedentary time with non-prolonged sedentary time, light and moderate-to-vigorous physical activity, ICAD

Additional Figure 7 legend:

Regression coefficients (95% CI) represent the difference in clustered cardio-metabolic risk excluding waist circumference when increasing that type of activity by 1 h/day while decreasing prolonged sedentary time by the same duration and holding other activity components constant. Differences in clustered cardio-metabolic risk excluding waist circumference when modelling 30 min/day substitutions instead of 1h/day substitutions (holding other activity components constant) equate to 50% of the estimates presented above. Models omitted prolonged sedentary time and incorporated non-prolonged sedentary time, light and moderate-to-vigorous physical activity and are adjusted for wear time (h/day), sex and age.
